# Amputation of Breast for Carcinoma; Pus Tubes and Ovaries Removed per Vaginam*Reported to the Louisville Clinical Society. Stenographically reported for this journal by C. C. Mapes, Louisville, Ky.

**Published:** 1898-11

**Authors:** William H. Wathen

**Affiliations:** Professor of Obstetrics, Abdominal Surgery and Gynecology in the Kentucky School of Medicine; Fellow of the American Gynecological Society and of the Southern Surgical and Gynecological Society; Gynecologist to the Kentucky School of Medicine Hospital and the Louisville City Hospital, etc., Louisville, Ky.


### Southern Medical Record.

A MONTHLY JOURNAL OF MEDICINE AND SURGERY.

VOL. XXVIII. ATLANTA, GA., NOVEMBER, 1898. NO. 11.

### Original Articles.

### AMPUTATION OF BREAST FOR CARCINOMA; PUS TUBES AND OVARIES REMOVED PER VAGINAM.*

By WILLIAM H. WATHEN, A.M., M.D., LL.D.

Professor of Obstetrics, Abdominal Surgery and Gynecology in the Kentucky School of Medicine; Fellow of the American Gynecological Society and of the Southern Surgical and Gynecological Society; Gynecologist to the Kentucky School of Medicine Hospital and the Louisville City Hospital, etc., Louisville, Ky.

This breast was removed five days ago from a large woman, otherwise healthy, about thirty years of age, married and the mother of one child six years old. She had called the attention of the family physician, Dr. J. W. Irwin, to the tumor about a week before I saw her, and had noticed its growth for more than a year. Her breasts were unusually large; the tumor was in the right breast above and on the inner side of the nipple; it was apparently about the size of a turkey's egg, but could not be distinctly outlined, penetrating imperceptibly into the surrounding tissues. The nipple was not involved, and the skin was not perceptibly changed except over the tumor, and even here it was not changed to the degree usually seen where cancerous tumors have reached this size, which was probably because of the great amount of fat intervening between the gland and the skin. The glands in the axilla could be felt enlarged.

She had been told that it would be necessary to excise but a little section of the breast, hence she was opposed to sacrificing her breast, but her wishes were not considered in the operation, and the entire breast was extirpated, with all fascia covering the pectoralis major muscle and extending down deep into the axilla. The axillary glands were found enlarged, varying in size from a French pea to the end of the

*Reported to the Louisville Clinical Society. Stenographically reported for this journal by C. C. Mapes, Louisville, Ky.

thumb, and several of them were so much degenerated that they had become caseous. After the axillary space had been cleansed of all infection by first dissecting the blood vessels and nerves from the glands which were removed with the fascia and fat, then by following the chain of lymphatics up to and under the clavicle, it was observed that the glands lying between the clavicle and the first rib in front of the vein were enlarged, ranging in size from a grain of wheat to a pea; these were enucleated from the vein and nerve and removed without dividing the muscles, by dissecting with the finger three or four inches underneath the tissues.

While the operation was thorough and all infected lymphatics removed, it could have been performed more easily and with less danger had we followed Halsted's method in removing nearly all the pectoralis major muscle and divided the pectoralis minor muscle, for then we could have exposed the infected lymphatic glands under the clavicle and have removed them without endangering vital structures so much as by enucleating the glands through an opening from the axillary space.

We assume that the patient will do as well without the removal of the pectoralis major and dividing the pectoralis minor as she would with it, for the reason that we removed the superficial surface of the muscle, and because of the fact that the lymphatics all go in the direction of the fascia and there is probably no involvment of the fascia behind the muscle.

*Cases* 2–3.—These specimens were removed from two patients with chronic pelvic peritonitis and tubal disease. In the first case we removed by vaginal section the left ovary and tube, the lumen of the tube being entirely obliterated. The tube and ovary on the right side were found adherent to the surrounding structures, but having separated them, they were brought into the vagina and found sufficiently healthy to return so that the woman might probably bear children. She was sitting up a week after the operation.

The second case was operated upon five days ago. She is a married woman, twenty-five years of age but has no children. Last summer while at a health resort in Wisconsin she had an acute attack of pelvic peritonitis. Returning to Chicago she consulted Dr. Etheridge who tried to cure her without a surgical operation. She came to Kentucky much improved under his treatment; and by keeping her bowels soluble, abstaining from sexual connection, observing general rules usual in such cases, she continued comparatively free from pain for several months; but when she began having sexual intercouse, taking exercise, etc., the pain returned, the exudations increased, and she was unfit for marital or domestic duties, so an operation became imperative. The uterus was about four inches in depth, with the endometrium in a septic condition; the cav ty was curetted from the fundus to the vagina; then an incision was made in the vaginal fornix into Douglas' pouch and the tubes and ovaries found adherent to everything adjacent. The adhesions were separated and the ovaries and tubes found so diseased that it was necessary to remove them. Clamps were put upon each side and removed after forty-eight hours.

When clamps are applied either in hysterectomy or in vainal incision, it is advisable to insert the finger above the bite and pack with gauze over the end of the clamp so that the intestines and omentum can not descend and become injured in the clamp. As many pieces of gauze may be packed in as may be necesssary. In removing the gauze it requires care, and if we have packed in several pieces we should not remove all of them at one time, but some pieces when the clamps are removed, then the following day other pieces are withdrawn allowing the tissues to close down gradually as we remove the gauze, and finally in four or five days it is all removed. Better protection is secured by this method; and we will cite an instance where the neglect of this precaution resulted in a condition that might frequently occur if we do not observe this method. We operated upon a patient at the Kentucky School of Medicine Hospital, removing an intraligamentous cyst per vaginam, and using gauze packing as described. My assistant removed the gauze at the end of four days, taking it all out at once. At the end of six days my attention was again called to the patient and she was found suffering intensely, with a large piece of omentum lying in the vagina so constricted in the opening as to require the separation of extensive adhesions around the thickened omentum before it could be pushed back into the abdominal cavity. As a result the woman had within ten hours a temperature of 1048 and pulse of 120. She recovered, but such conditions may result seriously. For this reason the gauze should be taken out gradually, and always insert the finger above to see that no omentum or coils of intestine are in the wound.

DISCUSSION.

*Dr. W. C. Dugan:* The first case reported is very interesting as all breast cases are. The Doctor stated there was no retraction of the nipple. This is not present in a large per cent. of cases, and it should not be looked upon as one of the symptoms of carcinoma of the breast. It is the exception rather than the rule, and you rarely find it unless you have a large schirrus cancer of the breast, which is seldom found in patients of the age stated. In schirrus where there is much connective tissue retraction of the nipple is common, but in the cellular form of cancers you rarely find it.

One question we should always inquire into, in these cases, which the reporter did not mention, is trauma. I have recently seen several cases of cancer of the breast, each one of them giving a history of trauma to which the growth could be directly traced. I believe this is a common exciting cause of cancer of the breast. It may be during lactation the child may bite the nipple or trauma from other causes may excite cancerous growth.

I believe in removing the breast in all suspicious tumors where the gland is involved. If you find the breast proper is not involved, then I believe you should simply remove the tumor and leave the breast intact, not mutilating the patient more than is absolutely necessary.

In regard to cleaning out the axilla: I have heard some surgeons contend that unless they could detect enlargement of the glands in the axilla before patient was put on the table they would not invade the axilla. It can not be denied that the glands are frequently infected without any apparent enlargement that can be detected before the axilla is opened. I have operated upon many cases of this kind, in which a careful examination beforehand, lifting up the patient's arm, running my finger along beneath the pectoralis major muscle and the chain of lymphatic glands I failed to find any enlargement whatever, when to my surprise after opening the axilla I found a number of enlarged and infiltrated glands. I take it that we should clean out the axilla in every case of carcinoma of the breast, and not only the glands but the adipose tissue. After dissecting the breast loose from its attachments, the chain of lymphatics leading from the gland to the axilla should be our guide.

One point that should be emphasized is, that in opening the axilla we should expose the vessels at the lower angle of the wound first and then dissect from below upwards for two reasons; first, it is easier to remove the glands and tissue in this way; second, if you are to expose the vein, and there is considerable danger from hemorrhage, if it is exposed from below upwards the hemorrhage can be controlled more easily. In all these cases I have made it a rule to expose the vessels at the lower angle of the wound first, then dissect from below upwards into the axillary space. If the patient's arm is lifted well up by an assistant so that the pectoralis major muscle may be easily recognized, you can dissect well up into the apex of the axilla without working much in the dark. This I have found by experience to be a great aid in the operation.

I have not found it necessary to perform the extensive myotomy that is practiced by Halsted and some others I believe it is unnecessary to remove the muscles unless you can trace the infection down into the muscle and find that the muscle is hardened by infiltration of cells, for, as Dr. Wathen states, the lymphatics run out from the muscles into the fascia rather than in the opposite direction.

The subclavicular chain of glands are very small, and must have been rather extensively involved or else the doctor would not have found any of them enlarged to the size of a pea. They must have been considerably hardened and infiltrated, for normal glands of that size would not be detected in the course of an operation.

In regard to vaginal ovariectomy: I have had but little experience in this method. I have operated only once or twice in that way for removal of the tubes and ovaries. I must say that I have not become wedded to that method of operating. I recognize the advantage of a tampon such as the doctor has described, leaving gauze intact so as to prevent herniation and prolapse of the pelvic viscera and omentum. In all the vaginal work I have done, which has been hysterectomy for carcinoma uteri, I have used the tampon, allowing it to remain intact for a week.

*Dr. T. P. Satterwhite:* I want to make reference to one point: We know that most patients dislike very much to have the breast removed; they suppose themselves to be practically unsexed on one side as far as destroying the appearance of the breast is concerned. Some years ago I had a similar case in an unmarried woman twenty-five years of age. She had a small tumor of the breast which did not give her any especial pain. It was about the size of a hickory nut. I removed this tumor and the tissues as I thought sufficiently far surrounding it In the course of six months it returned, and I told her I thought the breast had better be removed. She declined the operation. I told her the deformity would be less than she imagined, that I would leave the nipple. I dissected up the flap with the nipple, took out the tumor with the breast. The disease in the course of twelve months attacked the rectum and the patient eventually died of carcinoma recti. The deformity of the breast after the operation was very little, the opposite breast being rather small.

*Dr. W. H. Wathen:* I was pleased to hear Dr. Dugan call attention to the improved technique of these operations in beginning at the lower part of the axilla and dissecting from below upwards, because the work can be done more easily and successfully by that means.

Many doctors make the statement that if they can not feel the enlarged glands in the axilla, none exist, whereas there are many cases where it is impossible to feel a single enlarged gland, and when you open the axilla space thoroughly many glands will be found extensively involved. If you attempt to remove the glands there is no use stopping short of total extirpation of every one in the entire chain of lymphatics.

Probably one reason why some doctors believe that the glands are not always involved, even when the disease has existed for a considerable time, is that they confound the two diseases, carcinoma and sarcoma. Carcinoma if considered from its embryology, has its origin in tissues that arise from three blastodermic layers, the hypoblast, mesoblast and epiblast, hence the infection would be conveyed almost entirely through the lymphatics; whereas sarcoma arises in tissue having its origin solely from the mesoblast, hence the poison would be conveyed almost entirely through the veins. Therefore you might have very extensive sarcomatous growth without involvement of the axillary glands, but you will find very often that in sarcoma removed from the breast where there is no primary involvement nor secondary infection of the axillary glands that your patient may finally die from localization of the sarcomatous disease in some other parts of the body, apparently carried through the veins into the system, finally becoming localized at a point most favorable for its development. It is the exception to have axillary glandular involvement in sarcoma, and the absolute rule in carcinoma where the disease has extended to any considerable extent.

